# Supplementary figures and images for: Paclitaxel-Trastuzumab Mixed Nanovehicle to Target HER2-Overexpressing Tumors
Source: Nanomaterials (Basel). 2019 Jun 29;9(7):948. doi: 10.3390/nano9070948 (PMC6669497; doi:10.3390/nano9070948)

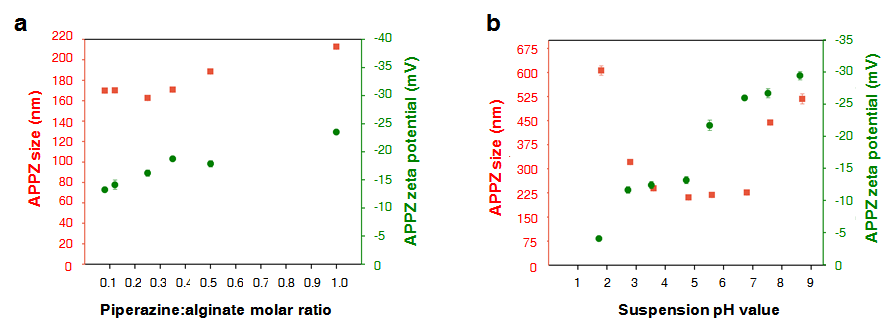

Supplement: Supplementary file 1 [file nanomaterials-09-00948-s001.zip › Supplementary files_rev/Fig. S1.tif]

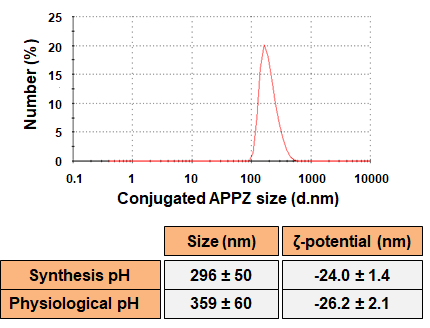

Supplement: Supplementary file 1 [file nanomaterials-09-00948-s001.zip › Supplementary files_rev/Fig. S2_rev.tif]

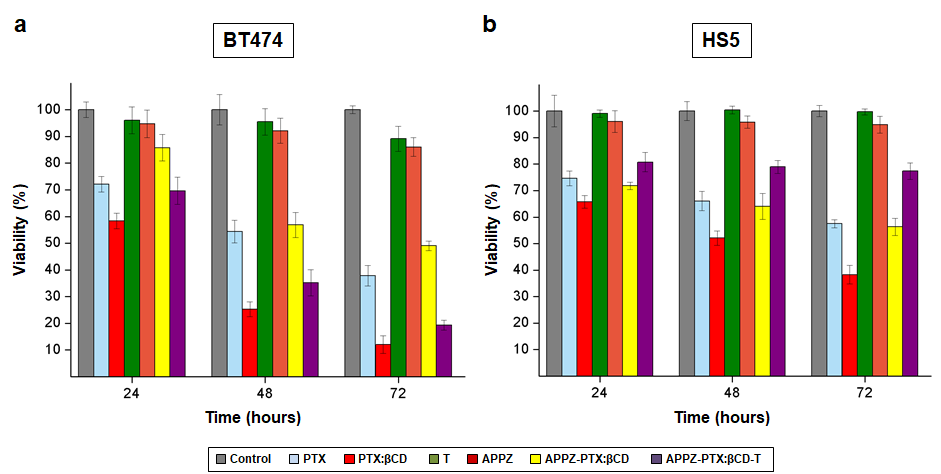

Supplement: Supplementary file 1 [file nanomaterials-09-00948-s001.zip › Supplementary files_rev/Fig. S3.tif]
